# Elevated Neutrophil‐to‐Lymphocyte Ratio Correlates With Liver Metastases and Poor Immunotherapy Response in Stage IV Melanoma

**DOI:** 10.1002/cam4.70631

**Published:** 2025-02-11

**Authors:** Yannick Foerster, Kristine Mayer, Sophia Wasserer, Marta Dechant, Vitalina Verkhoturova, Sarah Heyer, Tilo Biedermann, Oana‐Diana Persa

**Affiliations:** ^1^ Department of Dermatology TUM School of Medicine and Health München Germany

**Keywords:** checkpoint, immunotherapy, liver metastasis, melanoma, neutrophil‐to‐lymphocyte ratio, NLR

## Abstract

**Background and Objectives:**

Immune checkpoint inhibition (ICI) has revolutionized treatment for metastasized melanoma, but many patients remain unresponsive. Concerning potential adverse events, reliable biomarkers to predict ICI response are needed. In this context, neutrophil‐to‐lymphocyte ratio (NLR) and derived NLR (dNLR) have emerged. Liver metastases also limit ICI efficacy, correlating with diminished overall survival (OS) and progression‐free survival (PFS) and may siphon activated T cells from the systemic circulation, creating an ‘immune desert state’. We evaluated the predictive role of NLR and dNLR for ICI response and the impact of liver metastases on systemic immunity and treatment efficacy.

**Patients and Methods:**

In this single‐center retrospective study, we included 141 stage IV melanoma patients undergoing ICI. NLR and dNLR were calculated from absolute neutrophil count, absolute lymphocyte count, and white blood cell count.

**Results:**

Elevated NLR and dNLR were associated with poor response to ICI and inferior PFS. Patients with liver metastases exhibited higher NLR and dNLR levels and showed diminished response to ICI.

**Conclusions:**

Elevated baseline NLR and dNLR predict poor response to ICI and PFS in stage IV melanoma. Liver metastases are negative predictors for ICI response, with associated higher NLR and dNLR levels potentially contributing to therapy resistance.

## 1 Introduction

Immune checkpoint inhibition (ICI) through blockage of cytotoxic T‐lymphocyte‐associated protein 4 (CTLA‐4) and programmed cell death ligand 1 (PD‐L1) or anti‐programmed cell death receptor 1 (anti PD‐1) has emerged as a highly efficacious therapeutic approach for stage IV melanoma due to the high immunogenicity of this tumor entity. The combination therapy of ipilimumab plus nivolumab has demonstrated impressive 5‐year progression‐free (PFS) and overall survival (OS) rates of 36% and 52%, respectively [1]. Nonetheless, a notable proportion of patients remain unresponsive to ICI, leading to disease progression. While heightened PD‐L1 expression and tumor mutational burden (TMB) have shown associations with clinical response to ICI, their predictive accuracy remains somewhat limited [2, 3]. Therefore, there is a pressing demand for more robust biomarkers that are capable of predicting response to ICI. In this context, several parameters of systemic immune activity and inflammation have been explored, and recent studies have demonstrated that the neutrophil‐to‐lymphocyte ratio (NLR) and derived NLR (dNLR), which is calculated from absolute neutrophil count (ANC) and white blood cell count (WBC), may predict outcomes in patients with stage IV melanoma undergoing ICI [4, 5, 6].

It is also known that metastatic spread to the liver and the central nervous system (CNS) limits immunotherapy efficacy, correlating with diminished OS and PFS, respectively [7, 8, 9]. However, it remains unclear whether metastatic progression to these particular organs can impede systemic antitumoral immunity, thereby restricting ICI efficacy. There is evidence that liver metastases may eliminate CD8^+^ T cells from systemic circulation, which, in turn, may lead to an altered immune state in a preclinical model, thereby impeding ICI efficacy [10]. Moreover, liver metastases may induce local immunosuppression by infiltration of regulatory T‐cells and recruitment of CD11b^+^ monocytes [11, 12]. This is in line with the findings that irradiation of liver metastases augments ICI efficacy in preclinical models [13, 14], while also improving the response to ICI in patients with uveal melanoma [15].

In this study, we not only examined the predictive role of NLR and dNLR as biomarkers for response to ICI treatment in patients with stage IV melanoma, but also explored the potential impact of an altered immune state induced by liver metastases, which may manifest as elevated levels of NLR and dNLR.

## 2 Material and Methods

### 2.1 Patient Collective

A total of 141 patients with stage IV melanoma and available hematological values who received immune checkpoint inhibition (ICI) at our department between January 2017 and March 2024 were included in this study. Patients were selected for monotherapy or combination therapy based on their general condition, physician recommendations, and in some cases, patient preferences. Specifically, patients who were in poor general health or had concerns about the potential for serious immune‐related adverse events often opted for monotherapy. This selection process was influenced by both clinical judgment and patient choice. The therapy was administered either as the primary treatment or after disease progression. The following clinical data were collected: Gender, age at diagnosis, histological subtype, localization of the primary tumor, onset of disease, TNM classification, metastatic sites, onset of metastases, BRAF status, BRAF‐directed therapy prior or after ICI, response to BRAF‐directed therapy, duration of treatment, time of progression, time of death, tumor mutational burden (TMB), tumor proportion score (TPS), combined positive score (CPS). Hematological values including lactate dehydrogenase (LDH), absolute neutrophil count (ANC), absolute lymphocyte count (ALC) and white blood cell count (WBC) were collected within 6 weeks before starting treatment until last follow‐up or death. NLR and dNLR were calculated as NLR = ANC/ALC and dNLR = ANC/(WBC − ANC). To determine the cut‐off values for NLR, dNLR, ANC, and ALC, we conducted an analysis exploring all possible cut‐off points. We then selected the values that provided the best discrimination of progression‐free survival curves. Specifically, we maximized the *p*‐value in the log‐rank test to identify the cut‐offs that most effectively differentiated patient outcomes. This approach ensured that the chosen cut‐off points were associated with the most significant differences in survival. Over the course of treatment, tumor burden was monitored every 3 months by positron emission tomography‐computed tomography (PET‐CT) and magnetic resonance imaging (MRI) according to the national guidelines. Response to treatment was evaluated at least 3 months after administration of ICI. Progressive disease (PD) was defined as an increase in tumor burden assessed by PET‐CT or MRI. Complete response (CR) was defined as the disappearance of all tumorigenic lesions, and a partial response (PR) was defined as a 30% decrease in the size of tumorigenic lesions from baseline according to RECIST 1.1. Stable disease was defined as fitting the criteria neither for progressive disease nor PR. The majority of patients were treated with a combination of Ipilimumab and nivolumab for four courses followed by maintenance therapy with nivolumab unless unacceptable toxic effects or clear disease progression occurred. A minority of patients received nivolumab or pembrolizumab monotherapy.

### 2.2 Statistics

Progression‐free survival and overall survival were measured from the start of the treatment until tumor progression, death, or last follow‐up. *p*‐values for Kaplan–Meier curves were calculated by the log‐rank test. Hazard ratios and 95% confidence interval (95% CI) were calculated by using the Cox‐regression model. Means of NLR and dNLR at different times were compared by the unpaired *t*‐test. Observed and expected frequencies between two categorical variables were assessed by the chi^2^ statistic. *p*‐values lower than 0.05 were considered statistically significant (*p < 0.05, **p < 0.01, ***p < 0.001). All statistical analysis was performed by using SPSS statistics version 29.0.2.0 (IBM Corporation, New York, NY, USA) or R Studio 4.1.1. (Posit PBC, Boston, MA, USA). Images were created using the R package ggplot2. No imputations of missing values were made.

## 3 Results

Baseline characteristics of our patient cohort are presented in Table 1. Among our study population, 61.7% were male. Patient ages ranged from 20 up to 87 with a median of 63.1 years. The most common primary tumor site was localized on the trunk. Liver metastases were present in 31 patients, while 40 patients exhibited metastases in the CNS. The majority of patients (78.7%) received four courses of Ipilimumab plus nivolumab followed by nivolumab monotherapy. BRAF mutations were detected in 44.7% of patients, of which 52.4% received BRAF‐directed therapy either prior to or after ICI initiation. Median OS and PFS were 75 and 7 months, respectively. In our cohort, patients who received monotherapy had better PFS compared to those who received combination therapy (*p* = 0.025) (Figure S1). However, this difference was not statistically significant in the regression model.

**TABLE 1:** Baseline patient data. A total of 141 patients with stage IV melanoma and available hematological values who received immune checkpoint inhibition (ICI) at our department between January 2017 and March 2024 were included in this study.

Patient data	Total cases	%	Cases received combination	%	Cases received monotherapy	%
Total patients	141	100	111	100	30	100
**Gender**						
Male	87	61.7	68	61.3	19	63.3
Female	54	38.3	43	38.7	11	36.7
**Age (years)**						
Median	63.1		62		72.5	
Youngest	20		20		41	
Oldest	87		87		86	
**Primary tumor site**						
MUP	18	12.8	12	10.8	6	20.0
Head + Neck	20	14.2	15	13.5	5	16.7
Upper extremity	29	20.6	23	20.7	6	20.0
Lower extremity	29	20.6	23	20.7	6	20.0
Trunk	37	26.2	30	27.0	7	23.3
Mucosa	6	4.3	6	5.4	0	0
Missing	2	1.4	2	1.8	0	0
**M localization**						
Skin and/or lymphatic	23	16.3	10	9.0	13	43.3
Lung	27	19.1	20	18.0	7	23.3
Liver	31	22	28	25.2	3	10.0
CNS	40	28.4	36	32.4	4	13.3
Other	20	14.2	17	15.3	3	10.0
**Ulceration**						
Yes	74	52.5	59	53.2	15	50.0
No	33	23.4	26	23.4	7	23.3
Missing	34	24.1	26	23.4	8	26.7
**BRAF**						
V600E‐mutation	63	44.7	51	45.9	18	60.0
Wild Type	78	55.3	60	54.1	12	40.0
**LDH**						
Normal	78	55.3	58	52.3	20	66.7
Elevated	63	44.7	53	47.7	10	33.3
**Immunotherapy**						
Ipilimumab + Nivolumab	111	78.7	111	100		
Courses Combi ± SD (courses mono ± SD)	3.4 ± 1.4 (16.9 ± 13.3)		3.4 ± 1.4 (16.9 ± 13.3)			
Nivolumab monotherapy	9	6.4			21	70.0
Pembrolizumab monotherapy	21	14.9			9	30.0
Courses mono ± SD	21.8 ± 15.8				21.8 ± 15.8	
**BRAF‐directed therapy**						
Total	33	23.4	27	24.3	6	20.0
Prior to ICI (adjuvant)	3	2.1	2	1.8	1	3.3
Prior to ICI (palliative)	3	2.1	3	2.7	0	0
After ICI (palliative)	25	17.7	20	18.0	5	16.7
**Radiation therapy**						
Total	64	45.4	52	46.8	12	40.0
Prior to ICI (adjuvant)	14	9.9	10	9.0	4	13.3
Prior to ICI (palliative)	5	3.5	4	3.6	1	3.3
During ICI (palliative)	41	29.1	35	31.5	6	20.0
After ICI (palliative)	4	2.8	3	2.7	1	3.3
**OS (months)**						
Median [95% CI]	75 [34.7–115.3]		NR		75 [NA]	
**PFS (months)**						
Median [95% CI]	7 [4.3–9.7]		5 [2.8–7.1]		21 [0.0–51.2]	
**Follow‐up time (months)**						
Median [IQR]	21 [11.5–38]		19 [10–34]		35.5 [16.3–65.8]	
**Tumor mutational burden (TMB)**						
Total	27	19.1	24	21.6	3	10.0
Median [IQR]	7.9 [3.2–15.7]		7.2 [3.4–15]		21.4 [3.1–NA]	
**Combined positive score (CPS)**						
Total	66	46.8	62	55.9	4	13.3
Median [IQR]	3 [0–12.8]		3 [0–12]		10.5 [1–50]	
**Tumor proportion score (TPS)**						
Total	92	65.2	76	68.5	16	53.3
Median [IQR]	2 [0–9.5]		1.5 [0–6.5]		2 [1–25]	

Abbreviations: BRAF, mutation status of BRAF oncogene; CNS, central nervous system; IQR, interquartile range; LDH, level of serum lactate dehydrogenase; M, localization of distant metastasis; MUP, melanoma of unknown primary; NA, not applicable; NR, not reached; OS, overall survival; PFS, progression‐free survival; SD, standard deviation.

### 3.1 Elevated NLR and dNLR Are Associated With Poor Response to Immunotherapy and Inferior Progression‐Free Survival

**FIGURE 1:**
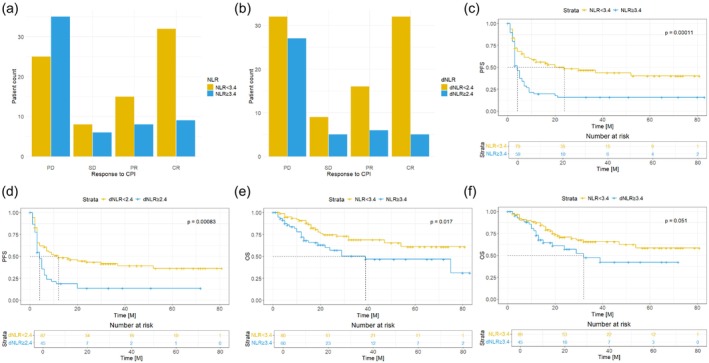
Response to treatment with immune checkpoint inhibitors (CPI). Progressive disease (PD) was defined as an increase in tumor burden assessed by PET‐CT or MRI. Complete response (CR) was defined as the disappearance of all tumorigenic lesions, and a partial response (PR) was defined as a 30% decrease in the size of tumorigenic lesions from baseline according to RECIST 1.1. Stable disease was defined as fitting the criteria neither for progressive disease nor PR. Patients with a neutrophil‐to‐lymphocyte ratio (NLR) below 3.4 (a) or derived NLR below 2.4 (b) showed significantly better response to CPI. NLR < 3.4 and dNLR < 2.4 were also associated with poorer progression‐free survival (PFS) (c, d) and overall survival (OS) (e, f).

We found that patients with a baseline NLR < 3.4 exhibited significantly better overall response rates (PR plus CR) to ICI compared to those with NLR ≥ 3.4 (*p* < 0.001) (Figure 1A). Similarly, patients with a cut‐off value of dNLR < 2.4 showed higher overall response to ICI compared to patients with dNLR ≥ 2.4 (*p* < 0.001) (Figure 1B). Furthermore, patients with a baseline NLR ≥ 3.4 experienced inferior median PFS after ICI initiation compared to those with a baseline NLR < 3.4 (*p* < 0.001) (Figure 1C). The hazard ratio for disease progression when comparing patients with NLR ≥ 3.4 to those with NLR < 3.4 was 2.149 (95% CI: 1.411–3.274). Similarly, patients with a dNLR ≥ 2.4 also showed higher risk for disease progression compared to patients with a dNLR < 2.4 (HR 1.967; 95% CI: 1.279–3.025), resulting in worse median PFS (*p* < 0.001) (Figure 1D). Similar results were observed for the subgroups of patients who received ipilimumab + nivolumab (Figure S2) and for patients who received only monotherapy (Figure S3).

Patients with an NLR ≥ 3.4 also turned out to have significantly worse OS compared to those with an NLR < 3.4 (*p* = 0.017) (Figure 1E). The HR for death during the observed time period was 1.982 (95% CI: 1.121–3.505). Additionally, patients with dNLR ≥ 2.4 showed a higher risk for death with an HR of 1.744 (0.971–3.131). However, this difference was not statistically significant (*p* = 0.051) (Figure 1F).

Vice versa, patients with NLR ≥ 3.4 had significantly higher response rates to BRAF‐directed therapy (*p* = 0.011) (Figure S4A). For dNLR, the response rate to BRAF‐directed therapy was statistically not significant (*p* = 0.125) (Figure S4B). No differences were observed between patient groups regarding TMB, CPS and TPS.

We also evaluated the impact of absolute neutrophil count (ANC) and absolute lymphocyte count (ALC) on response to treatment. We found that patients with an ALC exceeding a cut‐off value of 1.61 × 10^3^/μL showed significantly better overall response to ICI compared to patients below the cut‐off value (*p* = 0.006). In contrast, there were no differences regarding elevated ANC and response to ICI. Patients with ALC < 1.61 × 10^3^/μL exhibited significantly worse PFS compared to those with ALC ≥ 1.61 × 10^3^/μL (*p* = 0.003) (Figure S5A). Also, patients with ANC ≥ 5.05 × 10^3^/μL had significantly worse PFS compared to those with ANC < 5.05 × 10^3^/μL (*p* = 0.014) (Figure S5B). However, we found that neither ANC nor ALC were independent predictors for disease progression. When considering only patients with ALC < 1.61 × 10^3^/μL, we could not observe any differences regarding PFS and elevated ANC (Figure S6A). In contrast, when considering only patients with ALC ≥ 1.61 × 10^3^/μL, we found that patients with ANC ≥ 5.05 × 10^3^/μL exhibited significantly diminished PFS compared to those with ANC < 5.05 × 10^3^/μL (*p* = 0.012) (Figure S6B). Moreover, neither ALC nor ANC were associated with reduced or improved OS.

### 3.2 Liver Metastases Are Associated With Poor Response to Immunotherapy and Inferior Progression‐Free Survival

**FIGURE 2:**
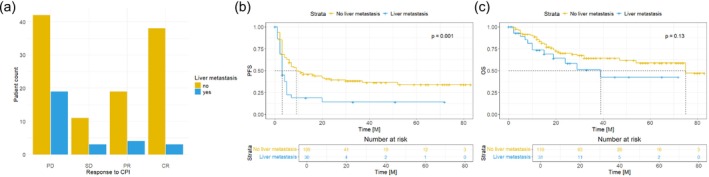
Response to treatment with immune checkpoint inhibitors (ICI). Progressive disease (PD) was defined as an increase in tumor burden assessed by PET‐CT or MRI. Complete response (CR) was defined as the disappearance of all tumorigenic lesions, and a partial response (PR) was defined as a 30% decrease in the size of tumorigenic lesions from baseline according to RECIST 1.1. Stable disease was defined as fitting the criteria neither for progressive disease nor PR. Patients exhibiting liver metastases prior to ICI administration showed significantly poorer responses to ICI (a). Liver metastases were also associated with poorer progression‐free survival (PFS) (b). Patients with liver metastases also exhibited poorer overall survival (c), but this difference was not statistically significant.

It is well‐known that liver metastases may restrain ICI efficiency. In our collective, patients who did not show evidence of liver metastases prior to ICI administration demonstrated a significantly higher overall response rate to ICI compared to those with pre‐existing liver metastases (*p* = 0.02). Specifically, only 27.6% of patients with liver metastases responded to ICI, while 51.8% of the patients without liver involvement exhibited an overall response to ICI (PR + CR) (Figure 2A). Moreover, clinical benefit (SD + PR + CR) was more prevalent in the group of patients lacking liver metastases (61.8% vs. 34.5%). Median PFS of patients without liver metastases was also significantly longer compared to those with liver involvement (*p*  0.001) (Figure 2B). The HR for disease progression was 2.079 (95% CI: 1.295–3.338), and 1.609 (95% CI: 0.834–3.105) for mortality. However, this difference was not statistically significant when considering mean OS (*p* = 0.13) (Figure 2C).

91.6% of patients with liver metastases showed a response to BRAF‐directed therapy compared to 60% of those lacking liver metastases (*p* = 0.054) (Figure S7).

Comparing patients with liver metastases with those lacking liver involvement, no significant differences were observed regarding TMB, CPS and TPS.

For the subgroup of patients lacking liver metastases, NLR < 3.4 and dNLR < 2.4 were both significantly associated with improved OS (NLR; dNLR: *p* = 0.008; *p* = 0.002) (Figure S8) and PFS (NLR; dNLR; *p* < 0.001; *p* = 0.002) (Figure S9), while patients with liver metastases showed a difference regarding elevated dNLR and inferior OS (Figure S10), but not PFS (Figure S11).

Furthermore, male gender, age over 70, elevated LDH level, presence of BRAF V600E mutation, and CNS metastases were associated with inferior PFS, whereas patients with pre‐existing lung metastases exhibited better PFS (Figure S12). Regarding OS, male gender, age over 70, elevated LDH and CNS metastases were associated with poorer OS. Patients with BRAF V600E mutation and lung metastases, on the other hand, showed favorable OS (Figure S13). HRs for progression and death depending on different parameters are presented in Table 2.

**TABLE 2:** Hazard ratios (HR) with 95% confidence interval (95% CI) for disease progression (top) and death (bottom) depending on different parameters.

Progression free survival	Hazard ratio (95% CI)		*p*
Male	1.169 (0.76–1.798)	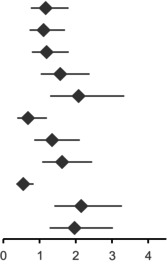	0.477
Age ≥ 70	1.111 (0.725–1.702)		0.63
BRAF V600E	1.193 (0.789–1.803)		0.402
LDH elevated	1.57 (1.036–2.38)		0.034
Liver metastasis	2.079 (1.295–3.338)		0.002
Lung metastasis	0.679 (0.384–1.201)		0.184
CNS metastasis	1.344 (0.855–2.111)		0.2
ANC ≥ 5.05	1.621 (1.072–2.451)		0.022
ALC ≥ 1.61	0.549 (0.361–0.833)		0.005
NLR ≥ 3.4	2.149 (1.411–3.274)		< 0.001
dNLR ≥ 2.4	1.967 (1.279–3.025)		0.002

Overall survival	Hazard ratio (95% CI)		*p*
Male	2.219 (1.104–4.464)	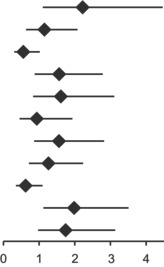	0.025
Age ≥ 70	1.148 (0.634–2.076)		0.649
BRAF V600E	0.557 (0.305–1.018)		0.057
LDH elevated	1.562 (0.876–2.783)		0.13
Liver metastasis	1.609 (0.834–3.105)		0.156
Lung metastasis	0.935 (0.453–1.931)		0.856
CNS metastasis	1.558 (0.861–2.822)		0.143
ANC ≥ 5.05	1.263 (0.714–2.232)		0.422
ALC ≥ 1.61	0.62 (0.351–1.095)		0.102
NLR ≥ 3.4	1.982 (1.121–3.505)		0.019
dNLR ≥ 2.4	1.744 (0.971–3.131)		0.063

Abbreviations: ALC, absolute lymphocyte count; ANC, absolute neutrophil count; CNS, central nervous system; dNLR, derived neutrophil‐to‐lymphocyte ratio; LDH, level of serum lactate dehydrogenase; NLR, neutrophil‐to‐lymphocyte ratio.

### 3.3 Patients With Pre‐Existing Liver Metastases Show Higher Levels of NLR and dNLR

**FIGURE 3:**
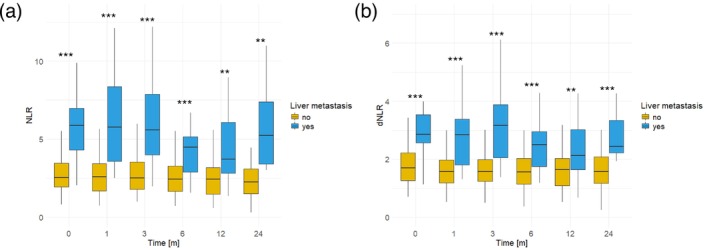
Neutrophil‐to‐lymphocyte (NLR) (a) and derived NLR (dNLR) (b) prior to and within 24 months (m) after start of immune checkpoint inhibition (CPI). Patients with liver metastases exhibited significantly higher levels of NLR and dNLR at all investigated time points (*p < 0.05, **p < 0.01, ***p < 0.001).

Previously, we demonstrated a negative correlation between both NLR and dNLR, as well as liver metastases, and the response to immunotherapy and median PFS following ICI initiation. To delve deeper into the impact of liver metastases on systemic immunity and the efficacy of ICI, our findings revealed a direct correlation between liver metastases and heightened levels of NLR and dNLR. Patients with liver metastases exhibited notably elevated levels of NLR and dNLR in comparison to those without liver involvement. This disparity was evident across various time points spanning 2 years following ICI initiation, including baseline measurements as well as assessments at 1, 3, 6, 12, and 24 months (NLR: 0 M, *p* < 0.001; 1 M, *p* < 0.001; 3 M, *p* < 0.001; 6 M *p* < 0.001; 12 M, *p* = 0.004; 24 M, *p* = 0.005; dNLR: 0 M, *p* < 0.001; 1 M, *p* < 0.001; 3 M, *p* < 0.001; 6 M, *p* < 0.001; 12M, *p* = 0.009; 24 M, *p* < 0.001) (Figure 3A,B). While CNS metastases are recognized as an additional site associated with a poor prognosis, our analysis did not reveal any discernible differences concerning NLR or dNLR (Figure S14). Furthermore, no distinctions were observed among different levels of NLR or dNLR concerning any other clinical parameter that is indicated in Table 1.

## 4 Discussion

Over the last decade, immunotherapy has revolutionized the landscape of melanoma treatment. However, there is a significant number of patients that fail to derive clinical benefit [16, 17, 18]. With concerns regarding potential serious adverse events [19] and the emergence of alternative therapies like BRAF‐directed drugs [20], there is a strong need for easily accessible biomarkers that can reliably predict responses to specific treatment options. In certain scenarios, such as when immunotherapy is paused due to serious immune‐related adverse events and disease progression occurs with no other treatment options, it is critical to evaluate the potential benefit of resuming therapy despite associated risks. Previous studies have shown a prognostic value of elevated levels of NLR among patients with a variety of solid tumors receiving ICI [21, 22, 23]. However, there is only very limited data available for patients with stage IV melanoma treated with ICI. Overall, we found two studies comprising a total of 322 patients that have shown an association between elevated levels of baseline NLR and reduced PFS and OS in patients with stage IV melanoma that were treated with ipilimumab + nivolumab followed by sequential monotherapy with nivolumab [24, 25]. In this studies, cut‐off values for NLR range between 4 and 5. However, we only found one study showing that elevated levels of baseline NLR and dNLR could predict the response to treatment with Nivolumab monotherapy in patients with metastatic melanoma [4].

In this study we analyze the association between baseline NLR and the response to treatment with ICI. The majority of patients received four courses of ipilimumab + nivolumab followed by nivolumab monotherapy, and few patients received nivolumab or pembrolizumab monotherapy due to reduced general condition or patient preference. We found that elevated levels of both NLR and dNLR were significantly associated with poor response to ICI. Moreover, NLR and dNLR were good predictors of PFS for patients with stage IV melanoma receiving ICI. In contrast, patients with high NLR exhibited significantly better response to BRAF‐directed treatment. However, these findings should be interpreted with caution, as they suggest that high NLR may serve as a prognostic parameter for patients receiving BRAF‐directed therapy. While these signals are promising, further validation in larger cohorts is needed.

The mechanisms underlying the association between a high baseline NLR and poor outcome of patients receiving ICI are poorly understood, but it is known that functional adaptive lymphocytes are important for antitumoral response in patients treated with ICI [26]. The number of tumor‐infiltrating lymphocytes assessed by different scoring systems, including TPS and CPS, are well‐established parameters to predict ICI response in patients with non‐small‐cell lung cancer (NSCLC) as well as head and neck squamous cell carcinoma (HNSCC) [27, 28]. In line with the results reported by Weide et al. [29], our study also demonstrated a direct correlation between the number of peripheral blood lymphocytes and the response to ICI, as well as PFS. Neutrophils, on the other hand, have direct and indirect protumoral and antitumoral effects during early stages of tumor initiation and growth [30]. Protumoral effects, for example, involve the release of reactive oxygen species (ROS) causing damage to DNA [31], support of tumor cell growth via various paracrine signaling pathways [32], or a modulation of the inflammatory tumor microenvironment (TME) by secreting PD‐L1 [33]. Antitumoral effects may include direct cancer cell killing [34] or the creation of an IFNγ‐rich TME to support antitumor activities of macrophages [35]. In this study, we observed that a higher ANC is associated with worse PFS, suggesting a more protumoral activity of neutrophils in melanoma, but ANC was not directly related to response to ICI. In addition, we found that a reduced peripheral lymphocyte count diminished the prognostic value of elevated peripheral blood neutrophils, whereas a peripheral blood lymphocyte count above the cut‐off value had no impact. These observations suggest a mutual reliance between ALC and ANC, emphasizing the superior prognostic efficacy of the composite predictors NLR and dNLR, respectively. As a systemic inflammation marker, NLR serves as an indicator of the equilibrium between immunosuppressive protumor neutrophils and adaptive antitumor lymphocytes.

We found that NLR and dNLR are robust and easily assessable predictive biomarkers for PFS and response to ICI independent from other clinical factors. Liver metastases also turned out to be independent negative predictors for the response to ICI in melanoma [7, 8, 9]. Consequently, we found that patients who did show evidence of liver metastases prior to administration of ICI had a significantly worse response rate to ICI compared to those patients who lacking liver metastases. This, in turn, also resulted in worse PFS in patients with pre‐existing liver metastases. The mechanisms underlying how liver metastases may impede the efficacy of ICI remain to be elucidated, but a recently published study by Yu et al. [10] in Nature Medicine could show that liver metastases siphon activated CD8^+^ T cells from systemic circulation. Within the liver, activated Fas^+^CD8^+^ T cells undergo apoptosis following their interaction with FasL^+^CD11b^+^F4/80 monocyte‐derived macrophages, creating a systemic ‘immune desert state’ in a mouse model. When considering our observations alongside the findings of Yu et al., we concluded that liver metastases may also be directly associated with a reduced NLR and dNLR, respectively. In fact, we are the first to report that patients with pre‐existing liver metastases exhibited significantly higher NLR as well as dNLR at all investigated time points prior to and within 2 years after ICI administration, which, in turn, may explain the poorer response to ICI of patients with liver metastases compared to those lacking liver metastases. Other clinical parameters associated with poor PFS, such as high levels of serum LDH and CNS metastases, did not correlate with elevated levels of NLR or dNLR, indicating a unique relevance of liver metastases for systemic inflammation homeostasis. However, in contrast to uveal melanoma [15], it is unclear if liver‐directed therapeutically approaches may enhance ICI efficacy in melanoma of the skin. A recent study by Lee et al. could show that liver metastases led to systemic suppression of antitumor immunity by a activation of regulatory T cells (Tregs) and modulation of CD11b^+^ monocytes which, in turn, reduced ICI efficacy. Remarkably, the systemic immunosuppression could be reversed by targeted depletion of Tregs, improving the therapeutic efficacy of ICI [12]. Interestingly, according to patients exhibiting elevated levels of NLR, those with liver metastases also seem to experience greater benefit from BRAF‐directed treatment.

The study has some methodological limitations due to its retrospective design and the heterogeneity among study participants. Hematological values were collected consistently across all treatment appointments to ensure data uniformity. However, the exact timing of collection prior to the start of treatment was heterogeneous. To account for this variability and obtain comparable values, we implemented a 6‐week window for data collection, acknowledging that this approach may introduce potential bias due to changes in the measured parameters over time. Additionally, a small number of patients received BRAF‐directed therapy prior to immunotherapy, which could introduce potential bias in our results. However, survival analysis was conducted using only the time from the start of immunotherapy. An additional notable finding is that patients receiving monotherapy exhibited better OS compared to those receiving combination therapy, but with only nine total events in the monotherapy group, this result may not be representative and should be interpreted with caution.

In conclusion, our study elucidates the significance of elevated baseline NLR and dNLR in predicting response to ICI and PFS. Optimal cut‐off values were 3.4 for NLR and 2.4 for dNLR, respectively. Our findings also underscore the importance of considering liver metastases as negative predictors for ICI response, with patients harboring liver metastases exhibiting higher NLR and dNLR levels, which may contribute to their diminished response to therapy. Moreover, our study is the first to provide insights into the potential benefits of BRAF‐directed treatment in patients with elevated NLR and liver metastases. However, further investigation into the mechanisms underlying the association between systemic inflammation, liver metastases, and treatment response is needed to optimize therapeutic strategies and improve outcomes for patients with metastatic melanoma.

## Author Contributions

**Yannick Foerster:** conceptualization (lead), data curation (lead), methodology (lead), visualization (lead), writing – original draft (lead). **Kristine Mayer:** writing – review and editing (equal). **Sophia Wasserer:** writing – review and editing (equal). **Marta Dechant:** writing – review and editing (equal). **Vitalina Verkhoturova:** writing – review and editing (equal). **Sarah Heyer:** writing – review and editing (equal). **Tilo Biedermann:** resources (lead), supervision (equal), writing – review and editing (equal). **Oana‐Diana Persa:** supervision (lead), writing – review and editing (lead).

## Ethics Statement

Reviewed and approved by the Ethics Committee of the Technical University Munich (2023‐103‐S‐KK). The patients in this manuscript have given written informed consent to the publication of their case details.

## Conflicts of Interest

The authors declare no conflicts of interest.

## Supporting information


Figures S1–S14

## Data Availability

The data that support the findings of this study are available from the corresponding author upon reasonable request.
