# Supplementary material for: Elevated Neutrophil‐to‐Lymphocyte Ratio Correlates With Liver Metastases and Poor Immunotherapy Response in Stage IV Melanoma
Source: Cancer Med. 2025 Feb 11;14(3):e70631. doi: 10.1002/cam4.70631 (PMC11811709; doi:10.1002/cam4.70631)
Supplement: Supplementary file 1 — Figures S1–S14 [file CAM4-14-e70631-s001.zip › Sup_Captions.docx]

Figure S1. Progression-free survival (PFS) and overall survival (OS) of patients treated with nivolumab or pembrolizumab monotherapy compared to patients treated with a combination of ipilimumab plus nivolumab.

Figure S2. Progression-free survival (PFS) and overall survival (OS) of patients treated with ipilimumab plus nivolumab, stratified by neutrophil-to-lymphocyte ratio (NLR) and derived NLR (dNLR).

Figure S3. Progression-free survival (PFS) and overall survival (OS) of patients treated with pembrolizumab or nivolumab monotherapy, stratified by baseline neutrophil-to-lymphocyte ratio (NLR) and derived NLR (dNLR).

Figure S4. Response to BRAF inhibitor therapy (BRAFi), stratified by baseline neutrophil-to-lymphocyte ratio (NLR) and derived NLR.

Figure S5. Progression-free survival (PFS) of patients treated with immune checkpoint inhibition, stratified by baseline absolute lymphocyte count (ALC) and baseline absolute neutrophil count (ANC) (x 10^3^ / μL).

Figure S6. Progression-free survival (PFS) of patients treated with immune checkpoint inhibition, stratified by baseline absolute neutrophil count (ANC) (x 10^3^ / μL). Only patients with a baseline absolute lymphocyte count (ALC) ≥ 1.61 x 10^3^/ μL were included.

Figure S7. Response to BRAF inhibitor therapy (BRAFi), stratified by the presence of liver metastasis.

Figure S8. Overall survival (OS) of patients without liver metastasis, stratified by baseline neutrophil-to-lymphocyte ratio (NLR) and derived NLR (dNLR).

Figure S9. Progression-free survival (PFS) of patients without liver metastasis, stratified by baseline neutrophil-to-lymphocyte ratio (NLR) and derived NLR (dNLR).

Figure S10. Overall survival (OS) of patients with liver metastasis, stratified by baseline neutrophil-to-lymphocyte ratio (NLR) and derived NLR (dNLR).

Figure S11. Progression-free survival (PFS) of patients with liver metastasis, stratified by baseline neutrophil-to-lymphocyte ratio (NLR) and derived NLR (dNLR).

Figure S12: Progression-free survival (PFS) depending on the presence of CNS metastasis, level of serum lactate dehydrogenase (LDH), BRAF status, age, gender and the presence of lung metastasis.

Figure S13: Overall survival (OS) depending on the presence of CNS metastasis, level of serum lactate dehydrogenase (LDH), BRAF status, age, gender and the presence of lung metastasis.

Figure S14: Neutrophil-to-lymphocyte (NLR) and derived NLR (dNLR) of patients with CNS metastasis prior to and within 24 months (m) after start of immune checkpoint inhibition.
