# Supplementary figures and images for: Elevated Neutrophil‐to‐Lymphocyte Ratio Correlates With Liver Metastases and Poor Immunotherapy Response in Stage IV Melanoma
Source: Cancer Med. 2025 Feb 11;14(3):e70631. doi: 10.1002/cam4.70631 (PMC11811709; doi:10.1002/cam4.70631)

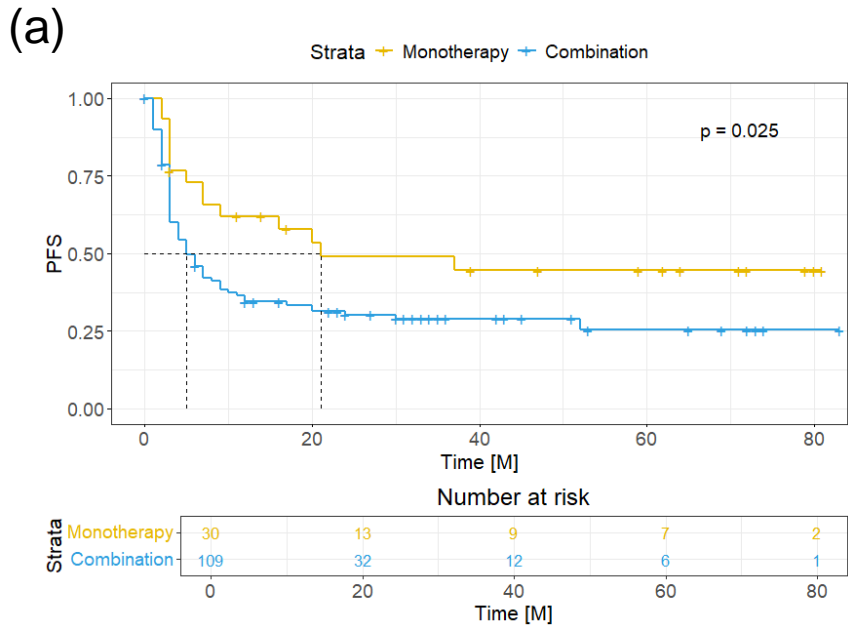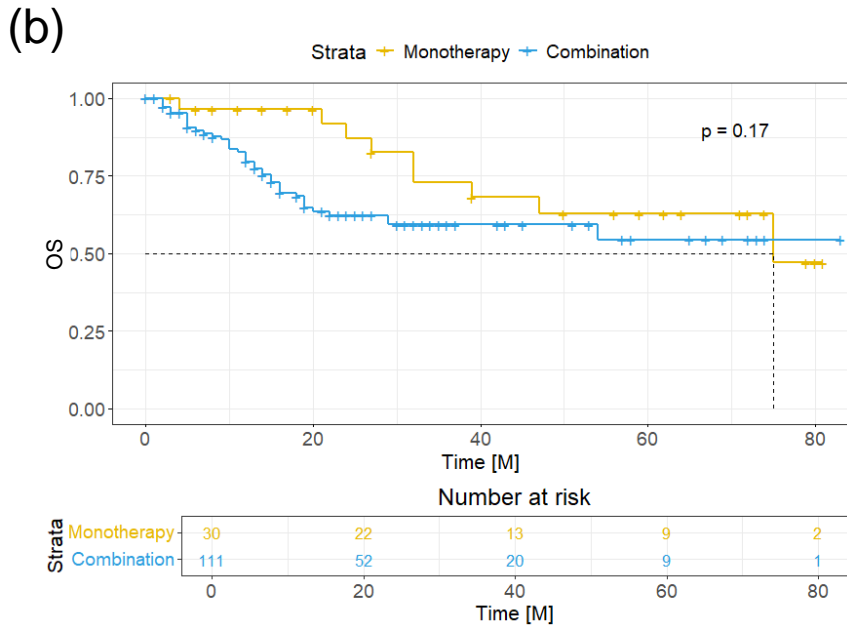

Supplement: Supplementary file 1 — Figures S1–S14 [file CAM4-14-e70631-s001.zip › FigureS1.pdf]

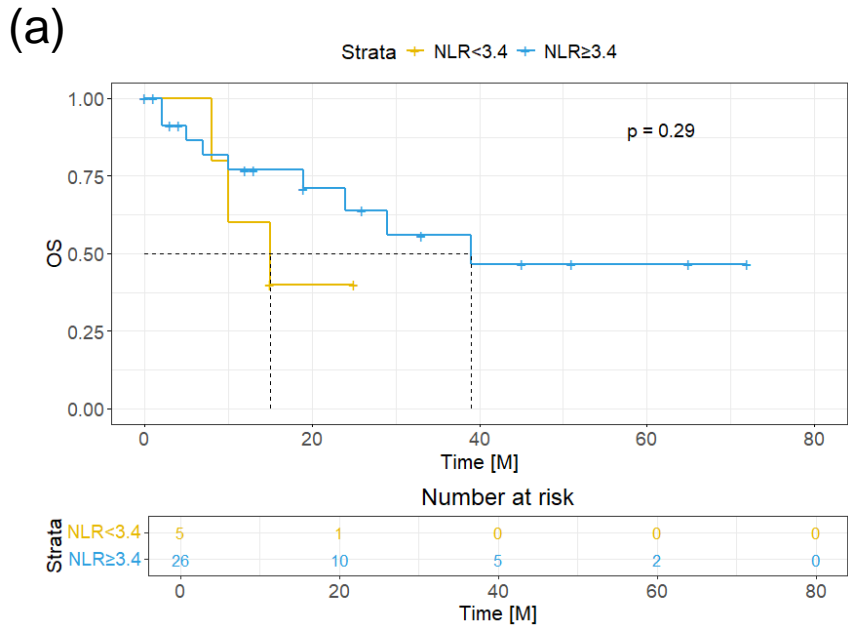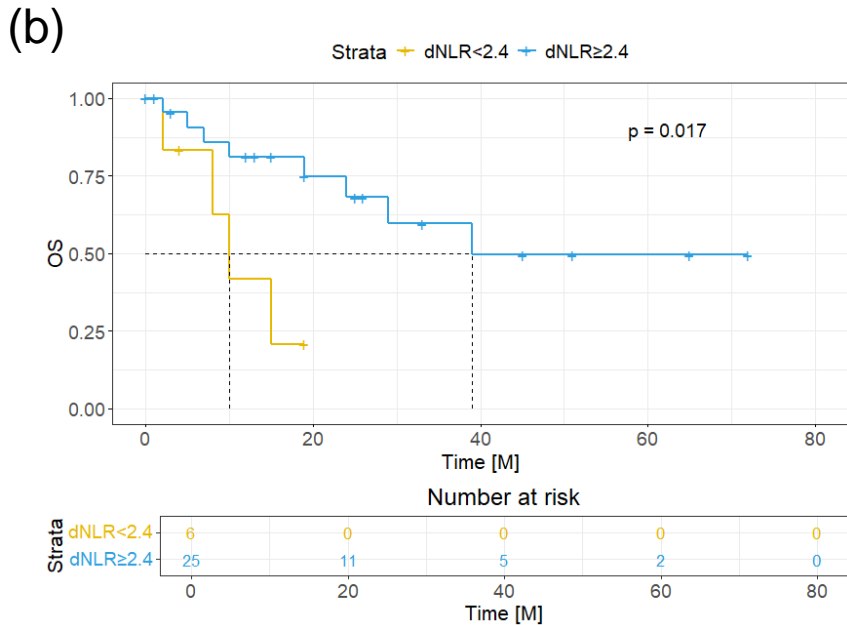

Supplement: Supplementary file 1 — Figures S1–S14 [file CAM4-14-e70631-s001.zip › FigureS10.pdf]

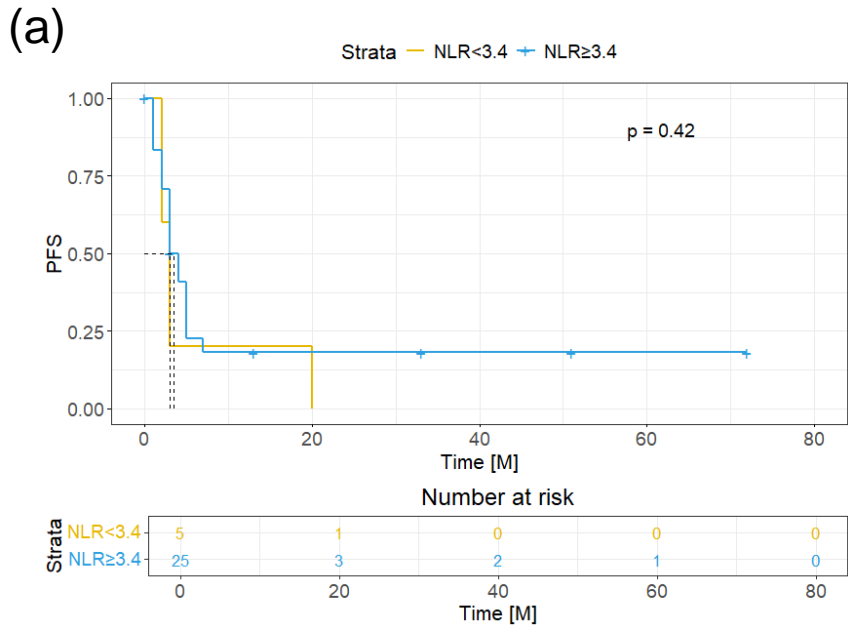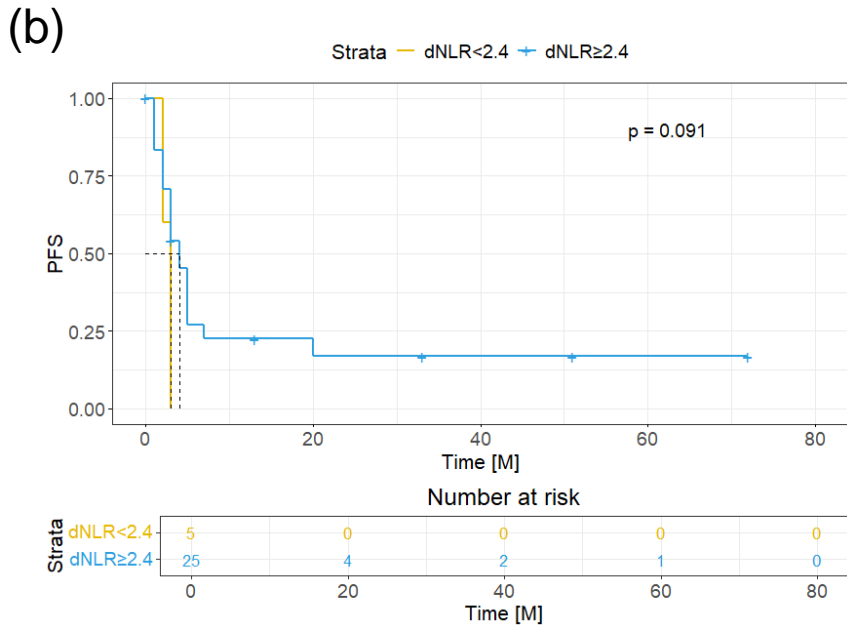

Supplement: Supplementary file 1 — Figures S1–S14 [file CAM4-14-e70631-s001.zip › FigureS11.pdf]

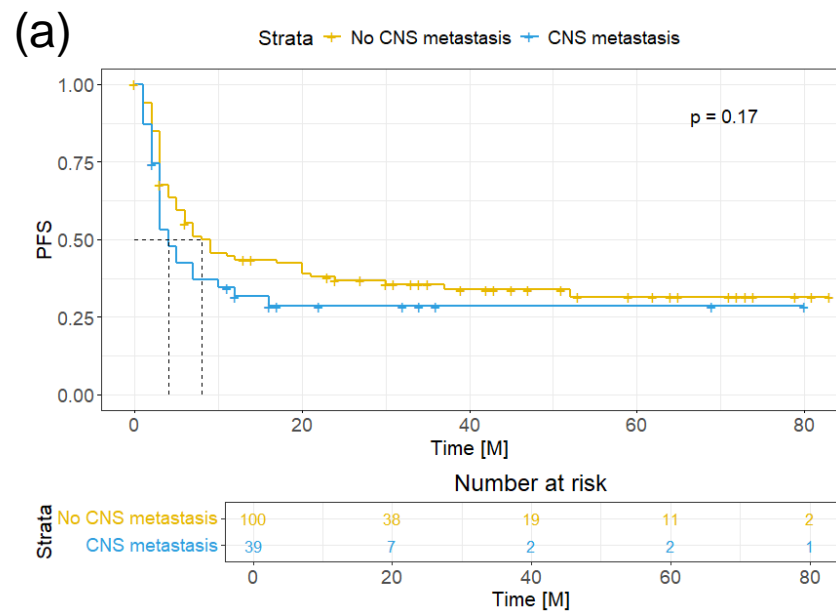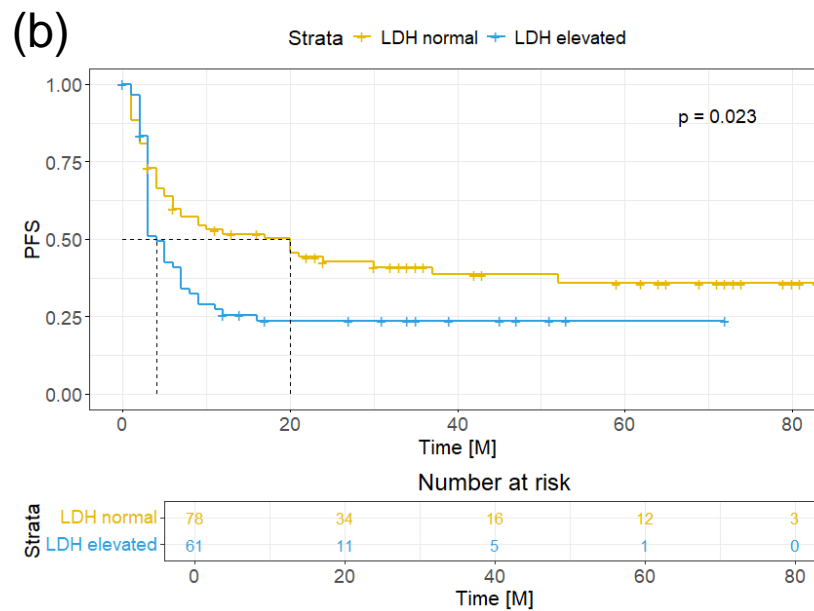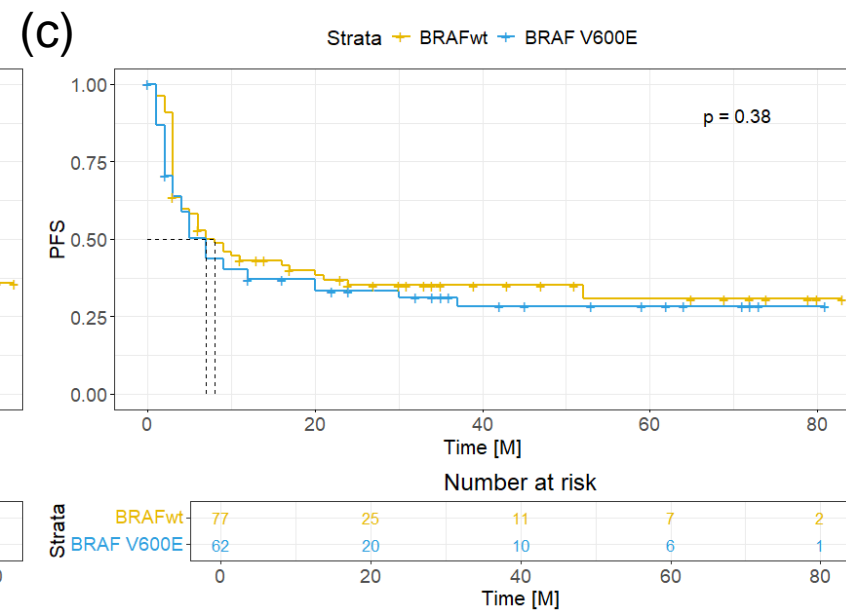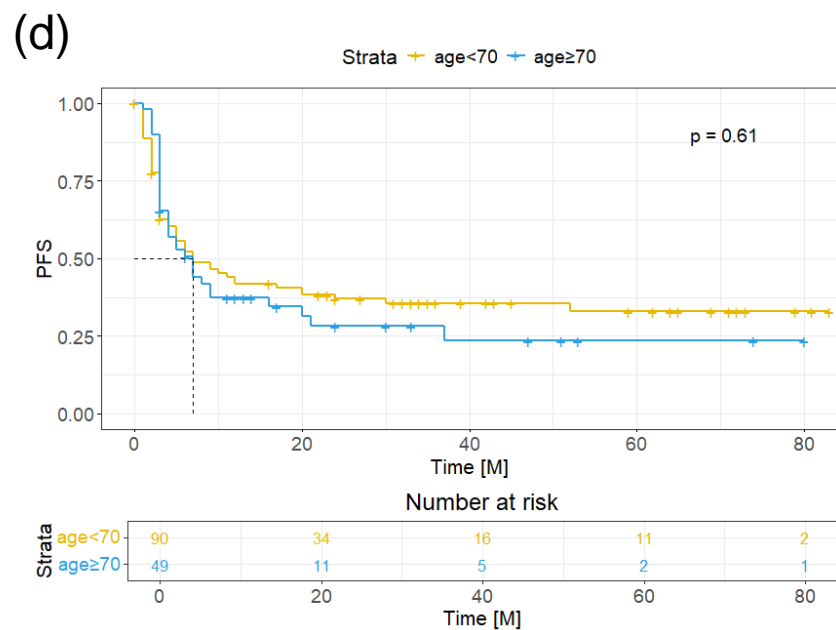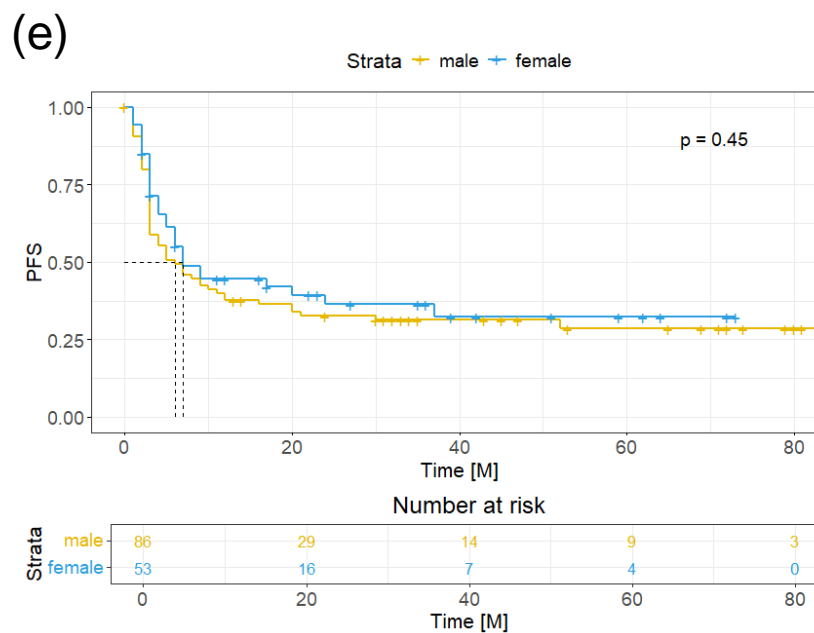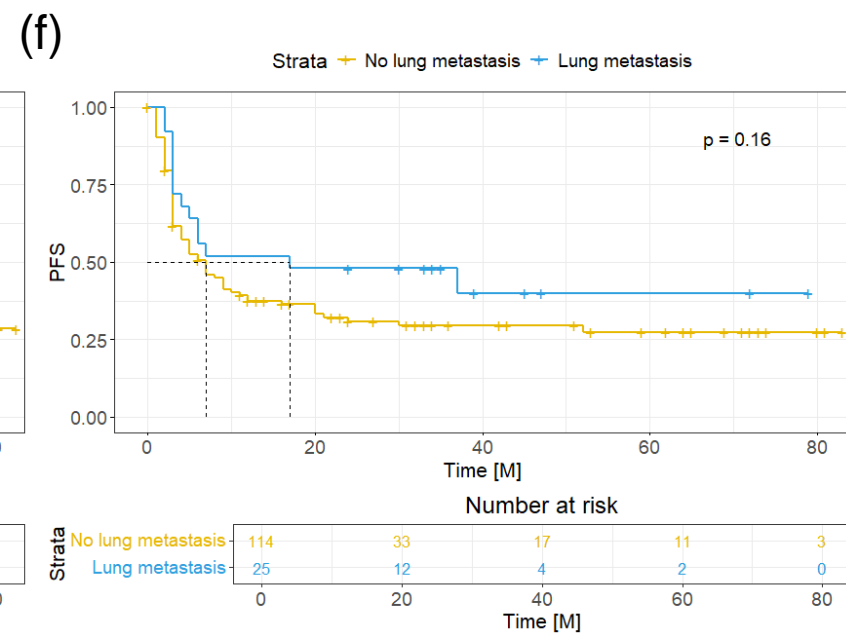

Supplement: Supplementary file 1 — Figures S1–S14 [file CAM4-14-e70631-s001.zip › FigureS12.pdf]

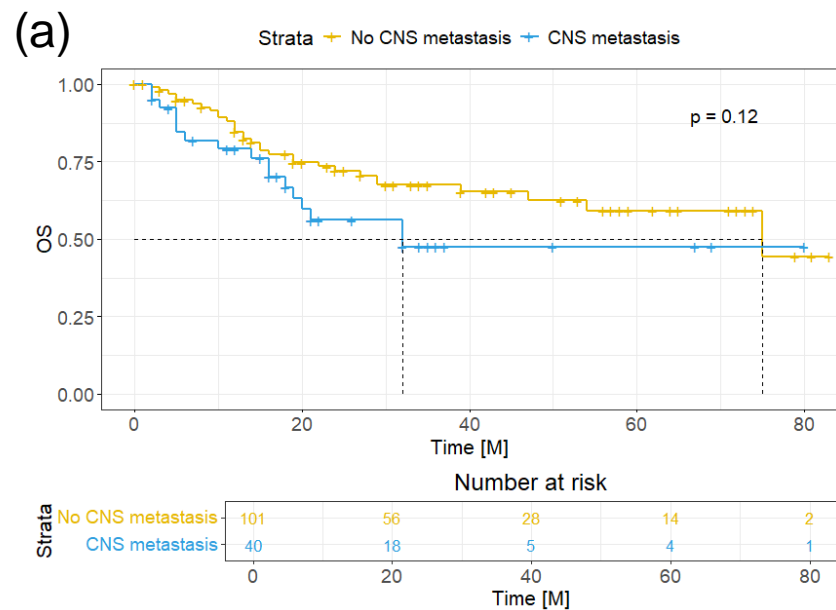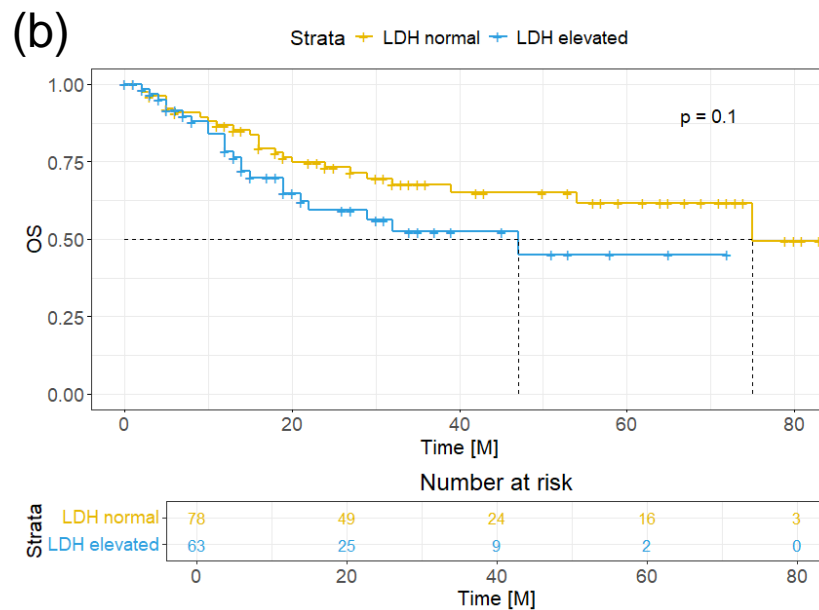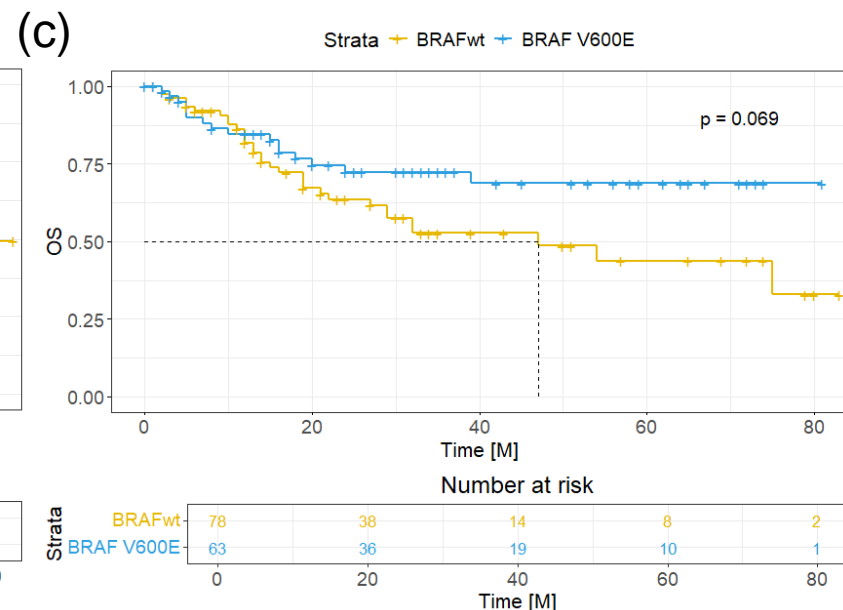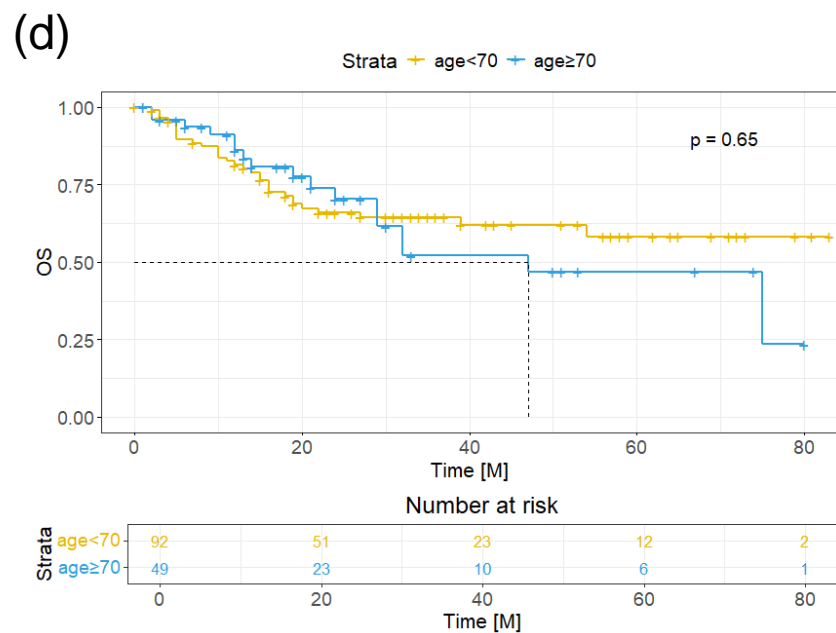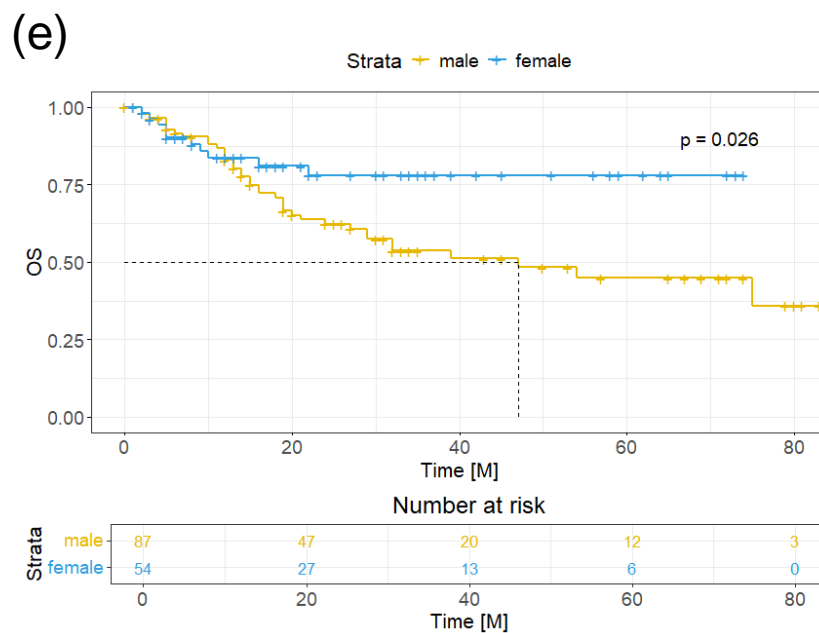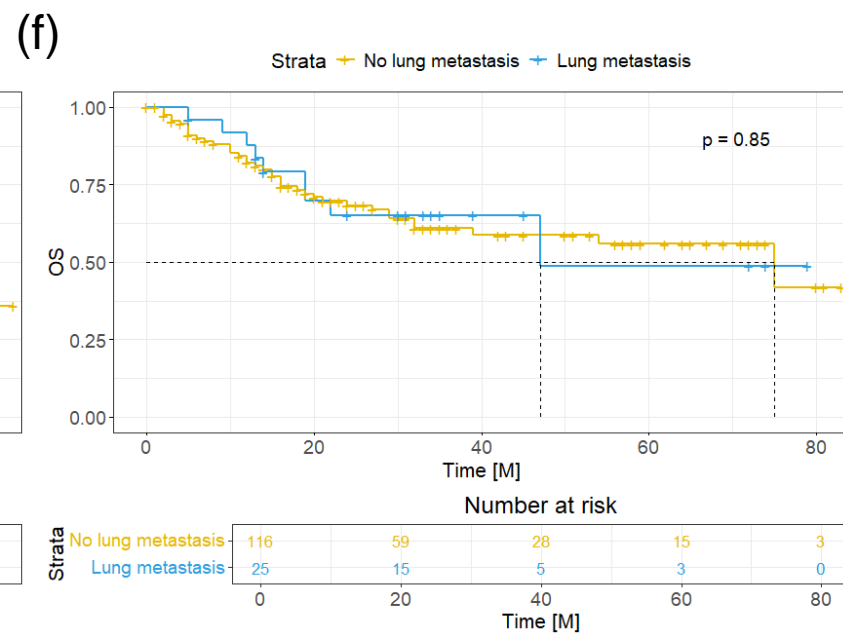

Supplement: Supplementary file 1 — Figures S1–S14 [file CAM4-14-e70631-s001.zip › FigureS13.pdf]

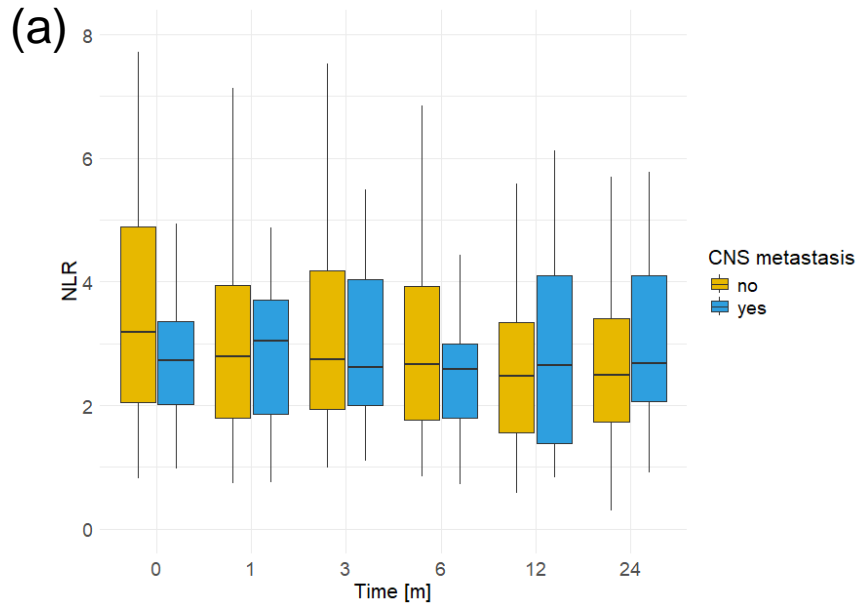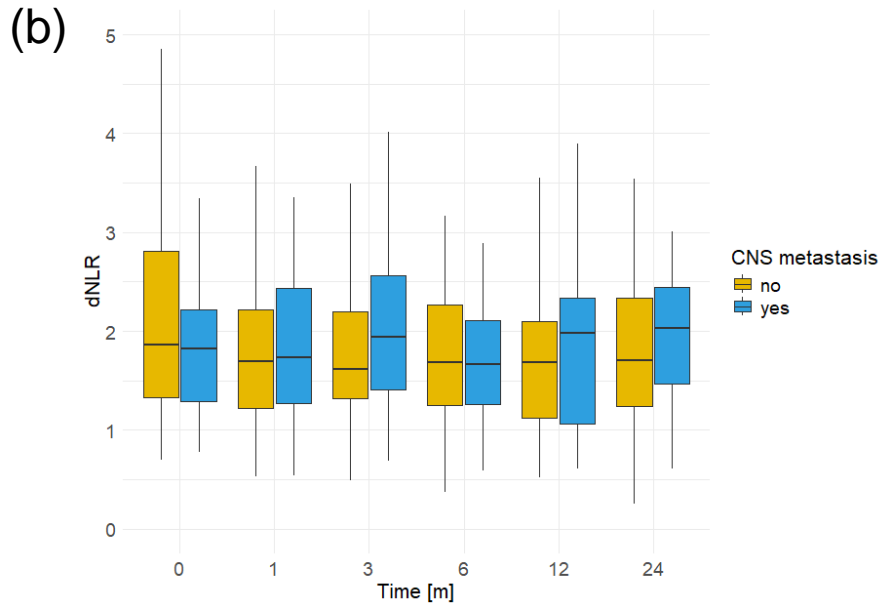

Supplement: Supplementary file 1 — Figures S1–S14 [file CAM4-14-e70631-s001.zip › FigureS14.pdf]

(a)

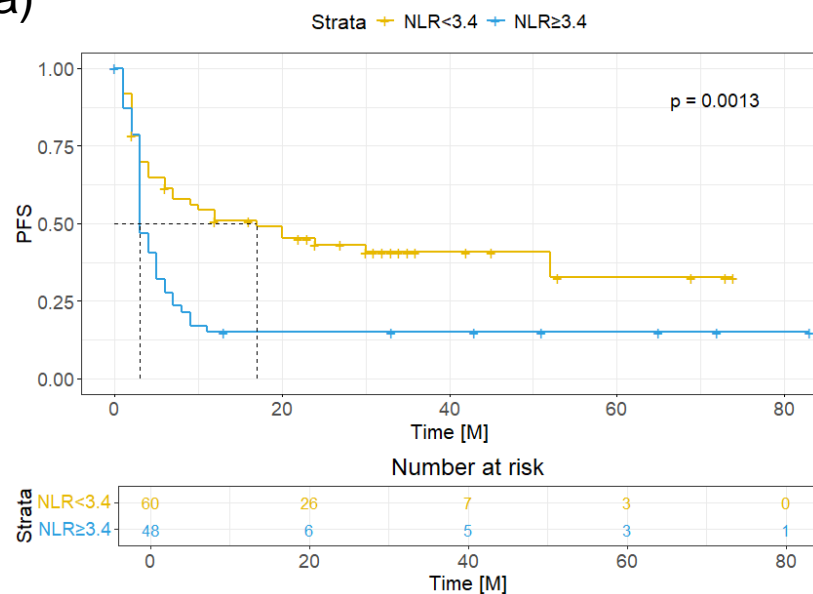

(b)

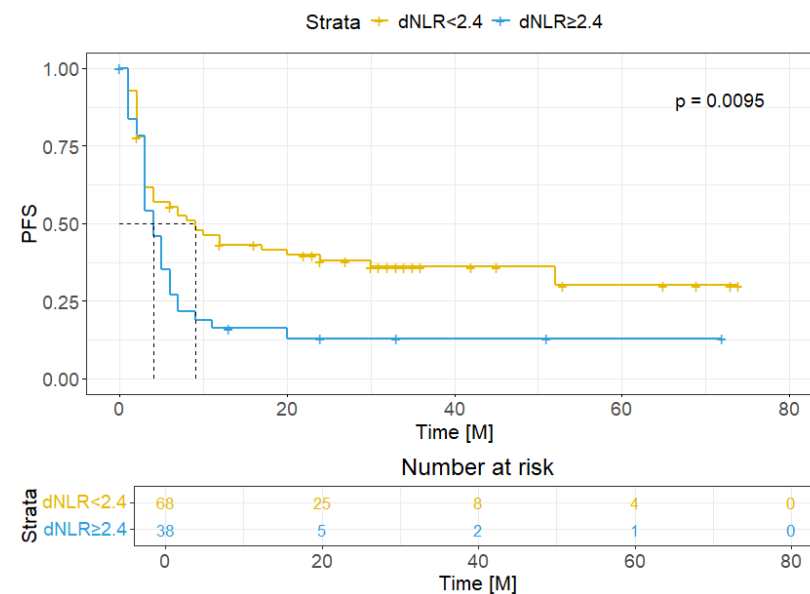

(c)

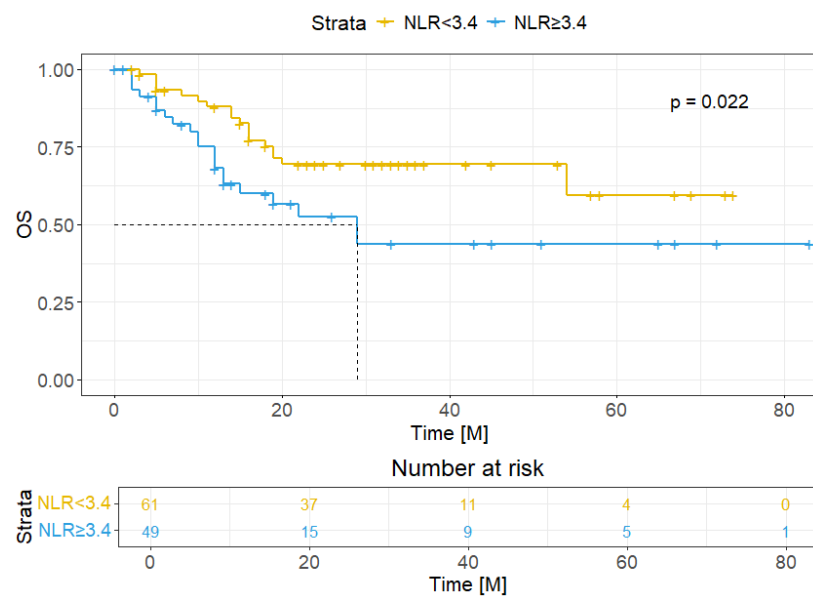

(d)

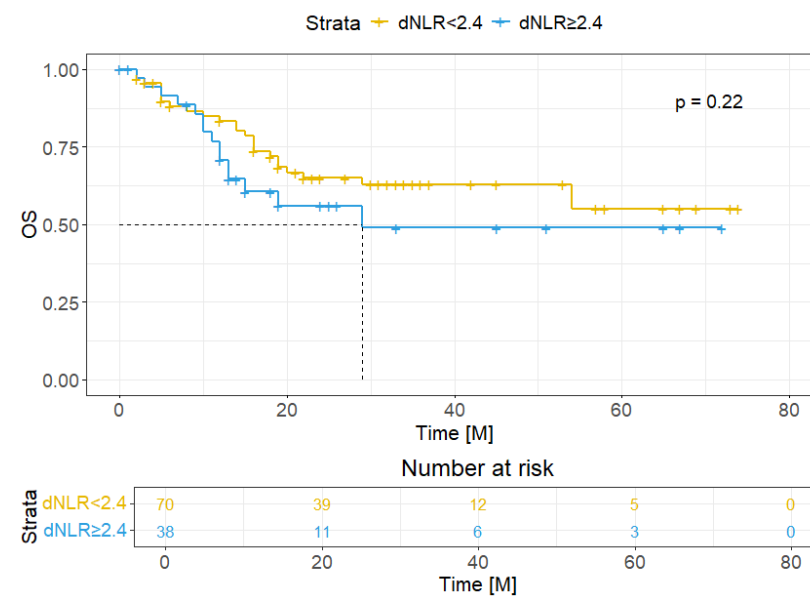

Supplement: Supplementary file 1 — Figures S1–S14 [file CAM4-14-e70631-s001.zip › FigureS2.pdf]

(a)

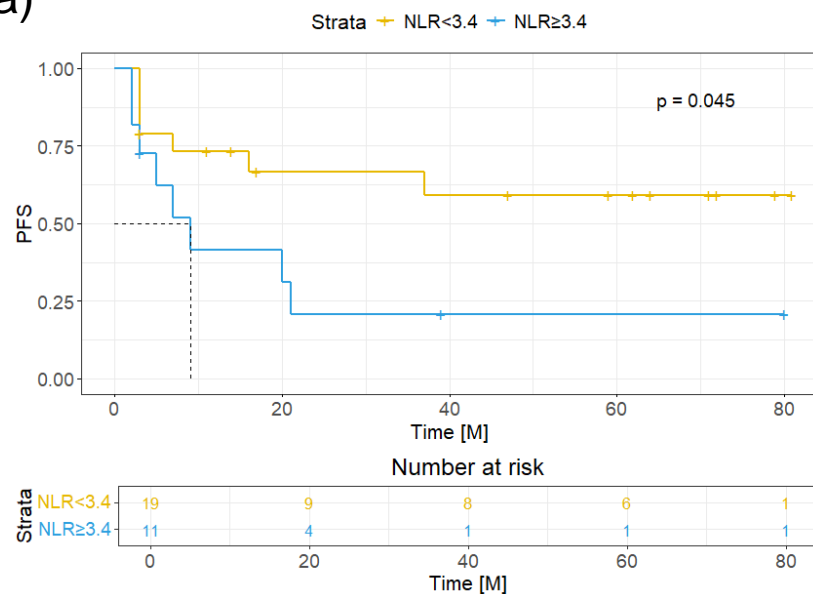

(b)

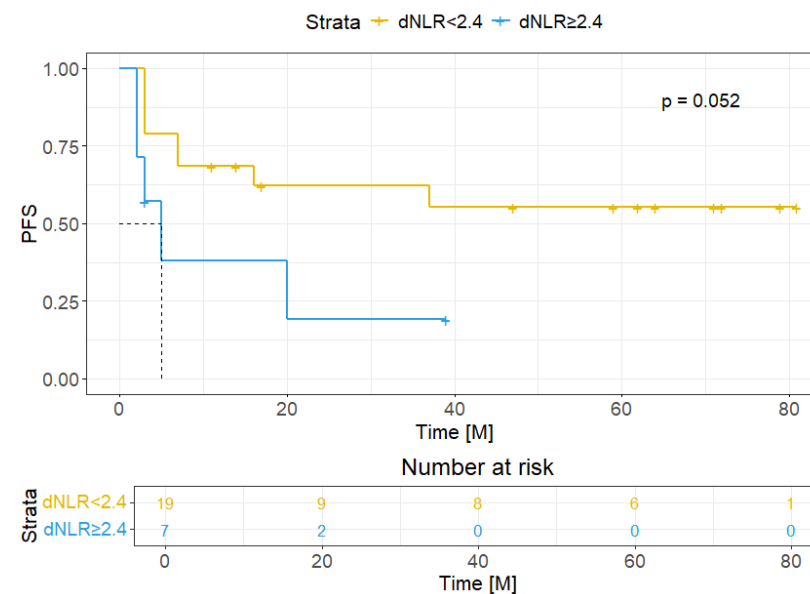

(c)

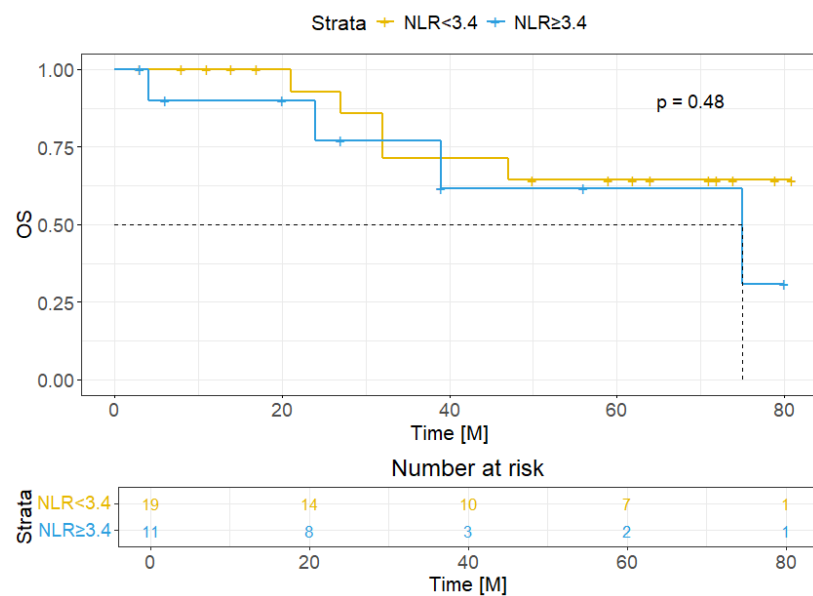

(d)

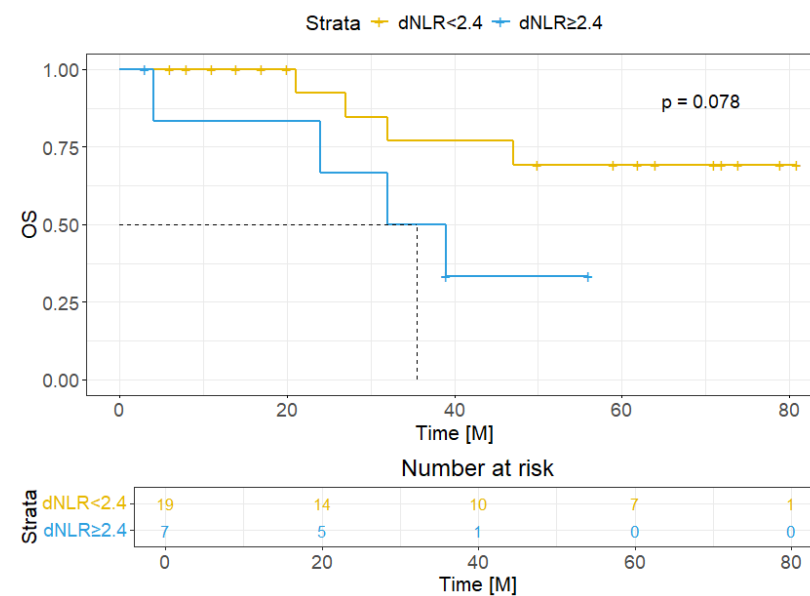

Supplement: Supplementary file 1 — Figures S1–S14 [file CAM4-14-e70631-s001.zip › FigureS3.pdf]

(a)

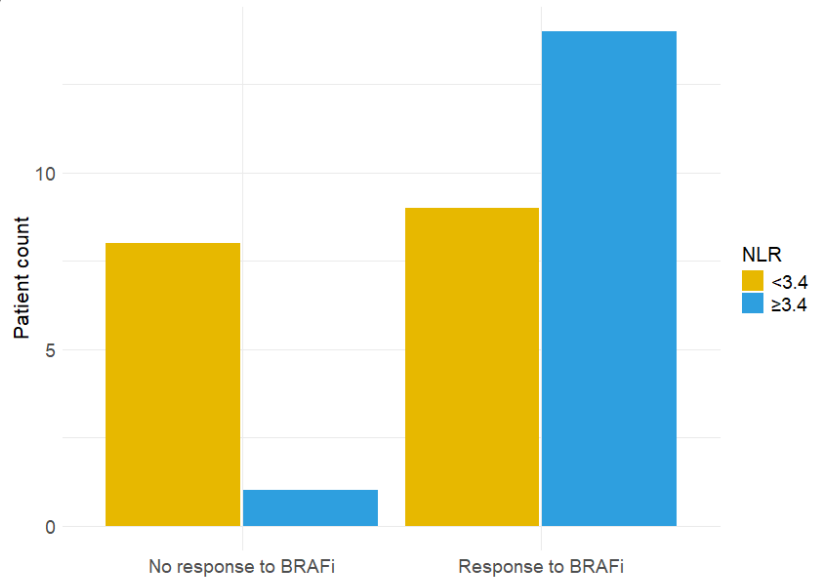

(b)

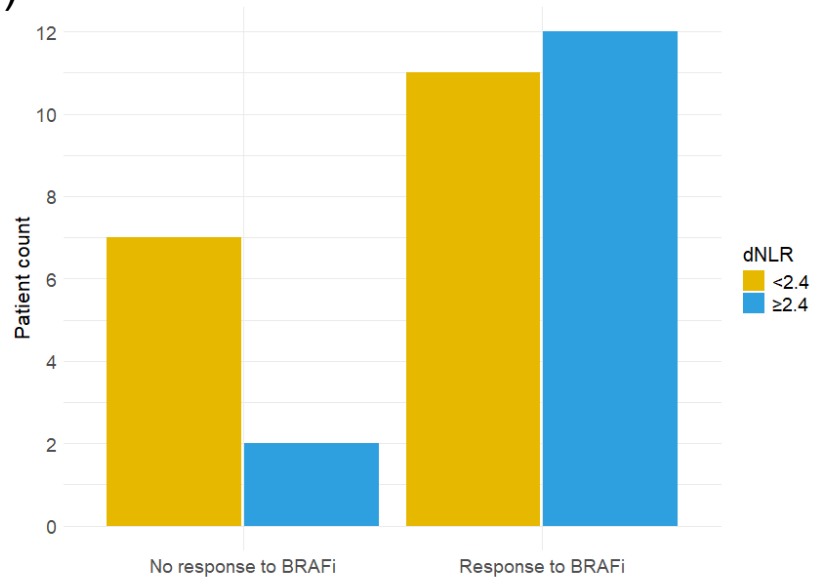

Supplement: Supplementary file 1 — Figures S1–S14 [file CAM4-14-e70631-s001.zip › FigureS4.pdf]

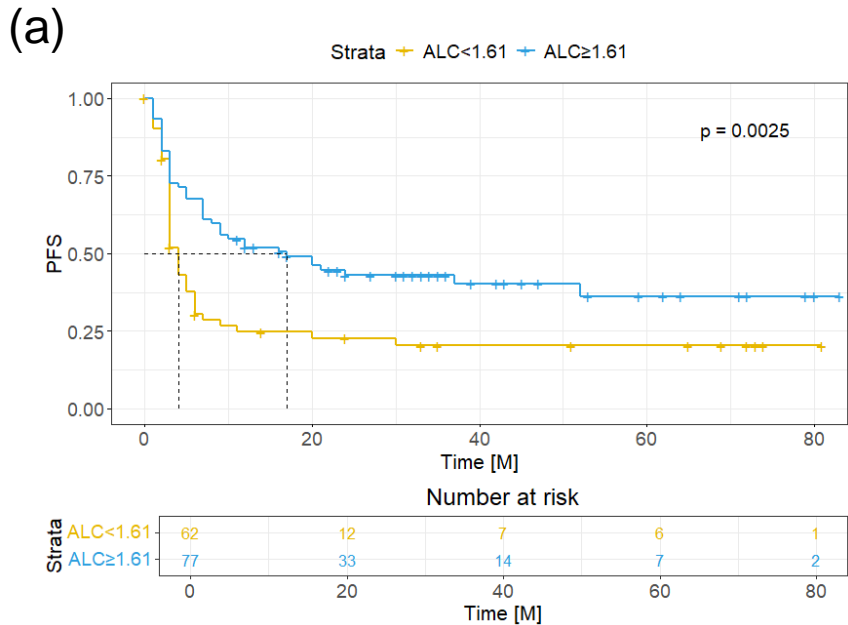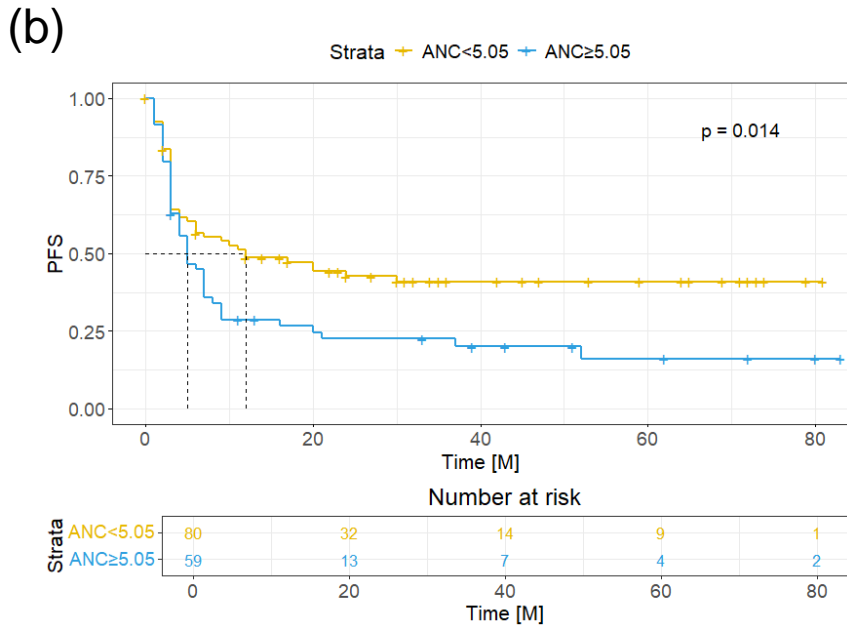

Supplement: Supplementary file 1 — Figures S1–S14 [file CAM4-14-e70631-s001.zip › FigureS5.pdf]

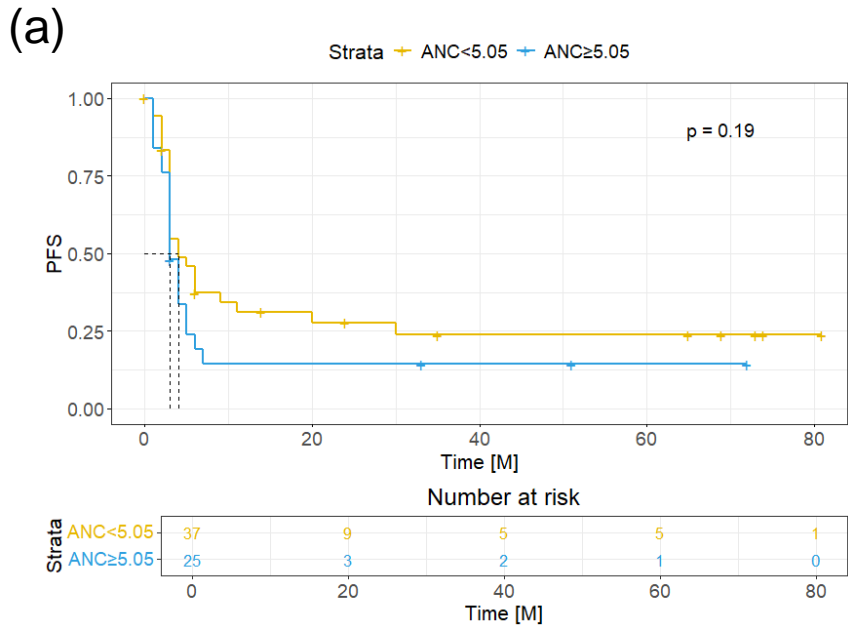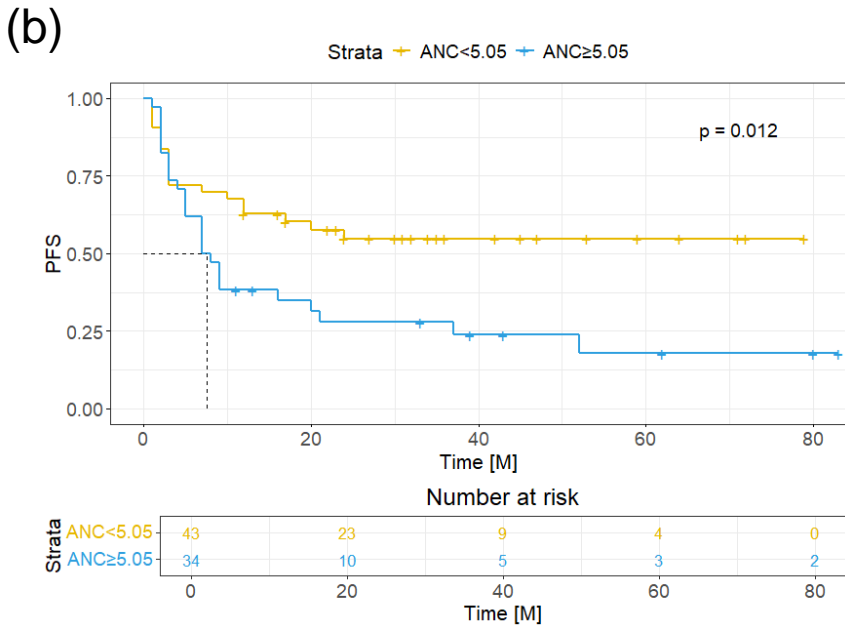

Supplement: Supplementary file 1 — Figures S1–S14 [file CAM4-14-e70631-s001.zip › FigureS6.pdf]

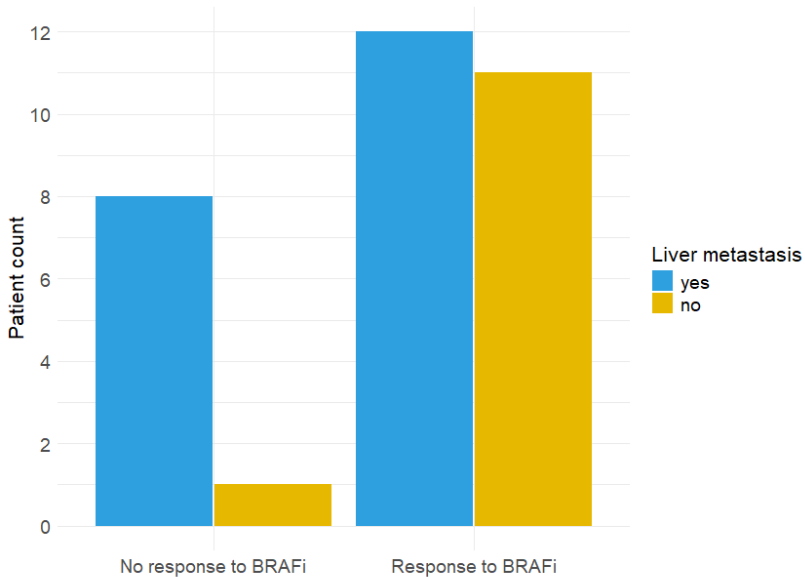

Supplement: Supplementary file 1 — Figures S1–S14 [file CAM4-14-e70631-s001.zip › FigureS7.pdf]

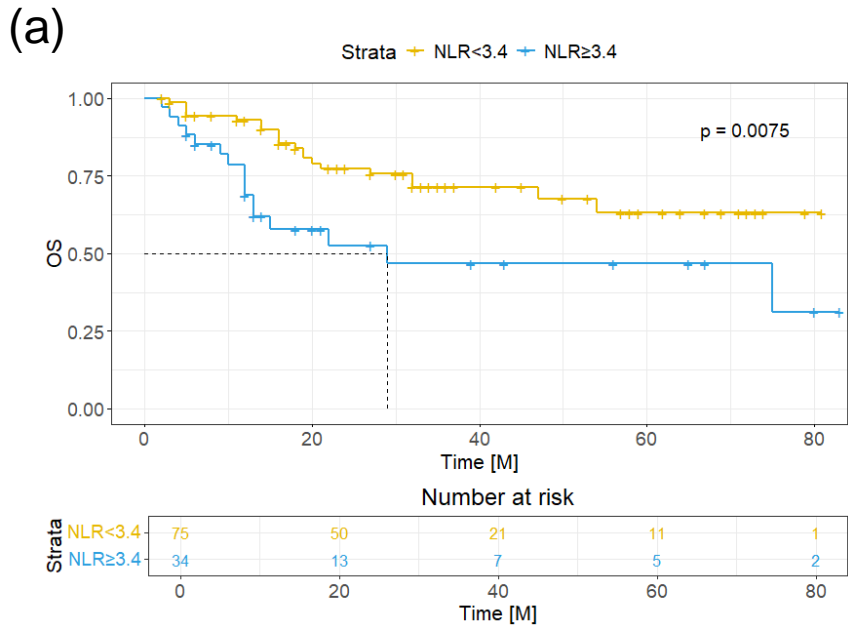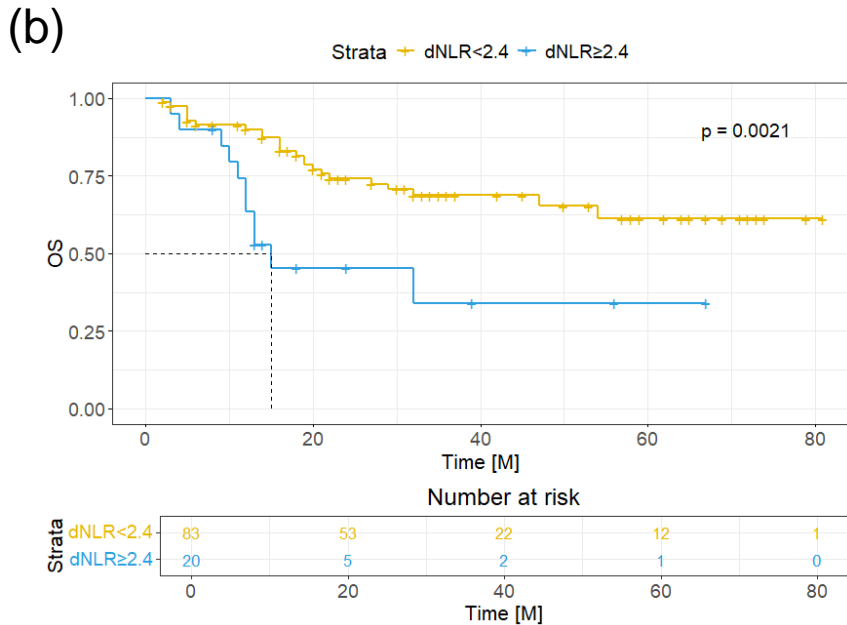

Supplement: Supplementary file 1 — Figures S1–S14 [file CAM4-14-e70631-s001.zip › FigureS8.pdf]

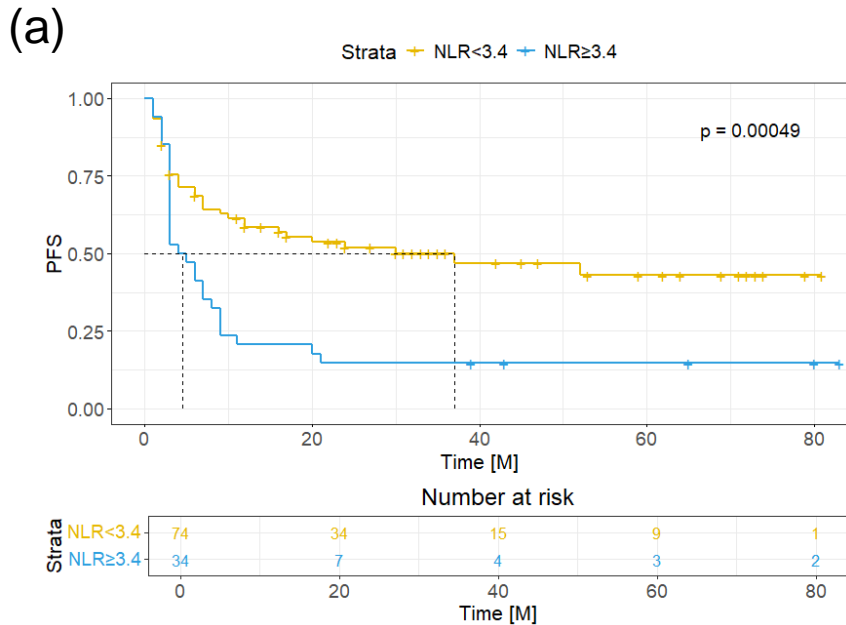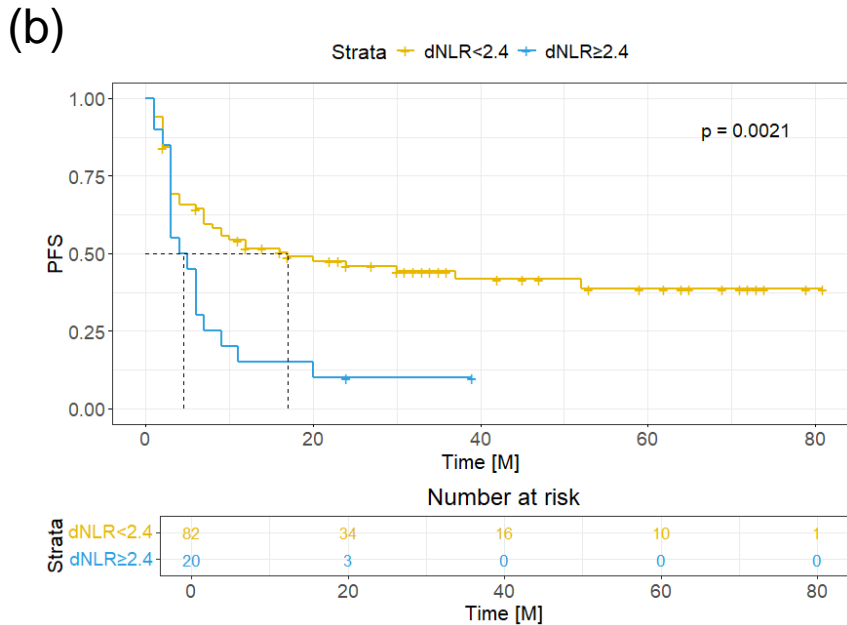

Supplement: Supplementary file 1 — Figures S1–S14 [file CAM4-14-e70631-s001.zip › FigureS9.pdf]
